# Closed Kinetic Chain Upper Extremity Stability test (CKCUES test): a reliability study in persons with and without shoulder impingement syndrome

**DOI:** 10.1186/1471-2474-15-1

**Published:** 2014-01-03

**Authors:** Helga Tatiana Tucci, Jaqueline Martins, Guilherme de Carvalho Sposito, Paula Maria Ferreira Camarini, Anamaria Siriani de Oliveira

**Affiliations:** 1 Department of Human Movement Science, Federal University of São Paulo, Santos, Brazil; 2 Department of Biomechanics, Medicine and Rehabilitation of Locomotor Apparatus, School of Medicine, University of São Paulo, Ribeirão Preto, Brazil; 3 Physiotherapy Orthopedic Clinic, Ribeirão Preto, Brazil; 4 Post-Graduation Program in Rehabilitation and Functional Performance, University of São Paulo, Ribeirão Preto, Brazil

**Keywords:** Upper extremity, Functional test, Reliability

## Abstract

**Background:**

The Close Kinetic Chain Upper Extremity Stability Test (CKCUES test) is a low cost shoulder functional test that could be considered as a complementary and objective clinical outcome for shoulder performance evaluation. However, its reliability was tested only in recreational athletes’ males and there are no studies comparing scores between sedentary and active samples. The purpose was to examine inter and intrasession reliability of CKCUES Test for samples of sedentary male and female with (SIS), for samples of sedentary healthy male and female, and for male and female samples of healthy upper extremity sport specific recreational athletes. Other purpose was to compare scores within sedentary and within recreational athletes samples of same gender.

**Methods:**

A sample of 108 subjects with and without SIS was recruited. Subjects were tested twice, seven days apart. Each subject performed four test repetitions, with 45 seconds of rest between them. The last three repetitions were averaged and used to statistical analysis. Intraclass Correlation Coefficient ICC_2,1_ was used to assess intrasession reliability of number of touches score and ICC_2,3_ was used to assess intersession reliability of number of touches, normalized score, and power score. Test scores within groups of same gender also were compared. Measurement error was determined by calculating the Standard Error of the Measurement (SEM) and Minimum detectable change (MDC) for all scores.

**Results:**

The CKCUES Test showed excellent intersession reliability for scores in all samples. Results also showed excellent intrasession reliability of number of touches for all samples. Scores were greater in active compared to sedentary, with exception of power score. All scores were greater in active compared to sedentary and SIS males and females. SEM ranged from 1.45 to 2.76 touches (based on a 95% CI) and MDC ranged from 2.05 to 3.91(based on a 95% CI) in subjects with and without SIS. At least three touches are needed to be considered a real improvement on CKCUES Test scores.

**Conclusion:**

Results suggest CKCUES Test is a reliable tool to evaluate upper extremity functional performance for sedentary, for upper extremity sport specific recreational, and for sedentary males and females with SIS.

## Background

Commonly, the musculoskeletal physical exam is focused on measures of range of motion (ROM) and muscular strength of the affected segment that could not provide enough information about overall segment functional level of activity [1]. Thus, shoulder evaluation could be improved by including techniques those assess functional movements and biomechanical impairment present in professional or daily life activities [1]. Functional tests could be considered a valuable complementary low-cost clinical tool to provide quantitative data about the functional ability and performance of a body segment [2,3]. Some tests also can be used to record the progress of a rehabilitation protocol by measuring, for example, performance and ability of a patient during physical task [4-7].

The *Closed Kinetic Chain Upper Extremity Stability Test* (CKCUES Test) is a performance test that provides quantitative data (score) for a upper extremity task in closed kinetic chain (CKC) with no needs of high technology to be realized in sportive or clinical settings. The test consists in counting how much times, during 15 seconds, the subject assuming a push-up position is able to touch his/her supporting hand with the swinging hand. The test is considered easy for clinicians to apply and also easy for clients to understand [8].

CKCUES Test was applied to subjects with different shoulder dysfunctions [8] and to evaluate shoulder performance before and after a muscle strengthening protocol [1,7]. However, the diversity of patients with shoulder dysfunction ranges from ordinary people who would like to return to their free-pain daily life activity to elite athletes that want to return to their professional routine [9]. As CKCUES Test is a performance test, subjects with low physical activity levels and patients with painful shoulder dysfunctions or injuries might find it difficult to properly perform or to complete the test, thus affecting score results and test reliability.

Number of touches reliability of CKCUES Test, in push-up position, was published only for a sample of recreational male athletes without shoulder dysfunction [2]. However, CKCUES Test can be performed by females in a modified (or kneeling) push-up position as suggested by original proposers and also can be used to assess males and females with shoulder conditions. Thus, to be considered as a tool to assess and follow upper extremity performance it is necessary to determine the test reliability considering genders, level of physical activity, and presence of shoulder impairment. Although, there was a variety of shoulder conditions those could be considered to be included in this study, a sample with subacromial impingement syndrome (SIS) was chosen for being the most common shoulder dysfunction causing pain and impairment of function [10-12].

Additionally, to apply CKCUES Test as a measure of shoulder performance improvement, it is important to know the minimal change without error that could be considered as meaningful to report clinically significant or real gain in the scores obtained between sessions [13,14]. Thus, the objectives of this study were (i) to determine the intrasession and intersession reliability of CKCUES Test scores for samples of sedentary healthy males and females, healthy recreational upper extremity sport specific athletes, and for sedentary males and females with SIS; (ii) to compare the scores of CKCUES Test among healthy sedentary, recreational upper extremity sport specific, and SIS samples and (iii) to determine the standard error of measurement (SEM) and minimum detectable change (MDC) of number of touches among groups to assist the clinical interpretation of shoulder performance improvement.

## Methods

### Subjects

The population of interest in this study was males and females with and without subacromial impingement syndrome. Subjects with no shoulder impairments were recruited from a local university area. Patients with SIS were recruited from a university’s orthopedic service. All participants read and signed consent form before starting in the study procedures. The study protocol was approved by the University Ethics Committee.

A total of 108 volunteers, aged between 20 and 65 years, were included in this study and their anthropometric data are described in Table 1. Volunteers were divided into six groups: a) healthy sedentary males (n = 20), b) healthy sedentary females (n = 20), c) healthy males’ upper extremity sport-specific recreational athletes (n = 20), d) healthy females’ upper extremity sport-specific recreational athletes (n = 20), e) sedentary males with SIS (n = 13) and f) sedentary females with SIS (n = 15).

**Table 1:** Demographic characteristic [mean and (standard deviation)] of volunteer groups

**Groups**	**Age (y)**	**Weight (Kg)**	**Height (m)**
**Sedentary male (n=20)**	24.95 (2.45)	81.99 (12.58)	1.75 (0.05)
**Sedentary female (n=20)**	22.65 (3.00)	56.25 (5.58)	1.62 (0.08)
**Active**^**¥ **^**Male (n=20)**	23.15 (2.48)	75.70 (10.49)	1.73 (0.05)
**Active**^**¥ **^**Female (n=20)**	21.75 (1.37)	57.45 (8.91)	1.61 (0.06)
**Male SIS**^**£ **^**(n=13)**	45.15 (12.59)	83.73 (12.73)	1.70 (0.06)
**Female SIS**^**£**^**(n=15)**	49.87 (5.87)	68.85 (15.87)	1.55 (0.06)

^¥^Active = young healthy male or female recreationally active for upper extremity sport-specific.

^£^SIS = subacromial impingement syndrome.

Subjects were considered sedentary when they performed less than 30 minutes of daily physical activity [15]. Additionally, the activities should be non-specific for upper extremity and without aim of physical training or competitions. Otherwise, subjects were considered physically active when they were performing one or more physical activity at least three times per week in the last 3 months or more, including at least one specific activity for upper extremity with no other reason than recreational fitness.

Inclusion criteria for SIS groups were history of shoulder pain for at least 3 months, at least two positive shoulder impingement tests (Neer, Hawkins/Kennedy, Jobe), shoulder abduction or flexion painful arc of motion (60°-120°); shoulder flexion and abduction of at least 90°, to be younger than 65 years-old; to be sedentary; and should not be in a physical therapy rehabilitation program. For healthy subjects, inclusion criteria were to be sedentary or upper extremity sport-specific recreational athletes, absence of shoulder pain; absence of previous surgery in the spine and upper extremity, and absence of complaints in the shoulder, elbow, wrist, hand and trunk musculoskeletal system.

Exclusion criteria for all volunteers were history of total tear in any muscle of shoulder complex; history of surgery or traumatic injury to the trunk, elbow or hand; history of luxation or orteoarthrosis in the glenohumeral or acromioclavicular joints; rheumatoid, neurological or degenerative disease; and positivity to Adison and/or Allen tests. Inclusion and exclusion criteria were checked through the interview and clinical examination performed by a physical therapist previously to the experimental procedure.

**Figure 1:**
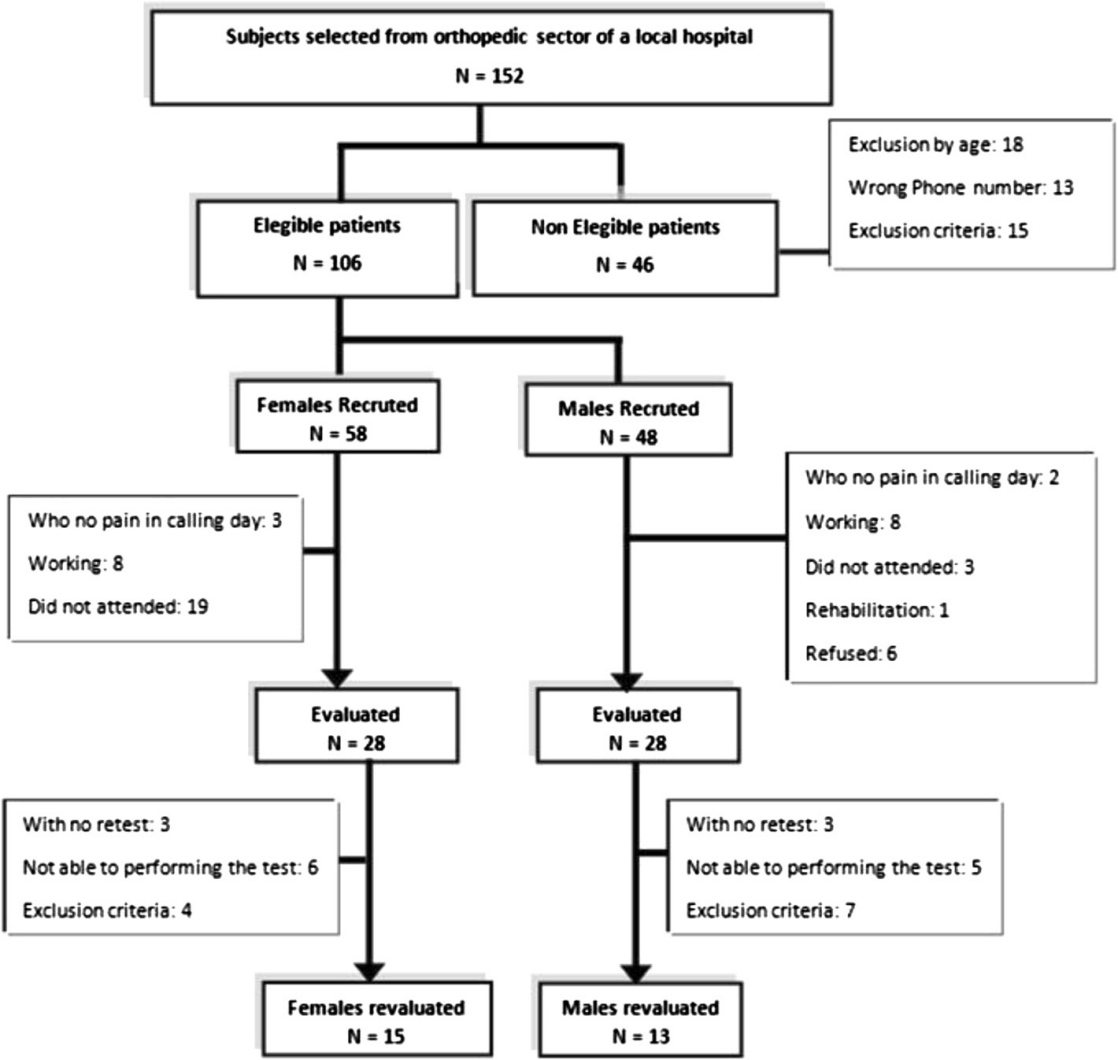
Subacromial shoulder syndrome (SIS) volunteer recruitment process flowchart.

A total of 152 medical records from orthopedic sector of a local hospital were analyzed and 106 patients with SIS diagnosed by a senior orthopedic physician were eligible for the study in a period of 11 months. From those 106 patients, 56 patients were recruited to perform the test, but 11 were unable to performing the test and 6 did not attend the retest day. Thus, 15 women and 13 men with subacromial impingement syndrome completed the study protocol (Figure 1). Clinical findings of the SIS group participants are briefly described. All subjects were positive for Neer and Hawkins’ tests and for painful arc of motion between 60° and 120°. Traumatic (unidirectional) joint instability tests were negative. SIS samples showed reduced values of active glenohumeral joint range of motion (ROM). However, they were able to rise their upper extremity at least 130° of shoulder flexion and 140° of shoulder abduction. Their grades for manual muscle testing (MMT) range from 5/normal (subject completes ROM against gravity with maximal resistance) to +3/fair plus (completes ROM against gravity with only minimal resistance). All MMT grades were reduced in the affected side of males and females when compared to the non-affected side, with exception of MMT grades for the shoulder extension muscle group which were reduced only in the female group.

### Testing procedure

As measuring the reliability of the CKCUES was one purpose of this study, test procedures were conducted by two examiners. The first examiner counted the number of touches. The second examiner was responsible to check the digital stopwatch and verbally informed the first examiner the beginning and ending of the test.

Two testing procedures were realized over 2 sessions, seven days apart. At the first session, the first examiner was responsible to explain how CKCUES test should be performed and, in the sequence, the examiner herself demonstrated the properly way to perform the test.

**Figure 2:**
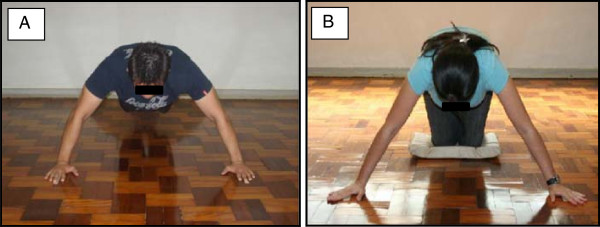
**CKCUES Test initial position. A.** Push-up initial position for male test, and **B.** Modified push-up initial position for female. Hands were 36-inches apart.

The CKCUES test is performed from a push-up position. Males perform CKCUES test by assuming a push-up position and females by assuming a modified (kneeling) push-up position; both with back flat parallel to the floor, hands at 36-inches apart and weight-bearing upper extremities positioned perpendicular to the floor and over the hands (Figure 2). Two parallel and aligned lines are marked on the floor to determine the initial placement of the hands. Thus, to beginning the test, subject assumes a push-up position with one hand of each line marked at the floor. Then, during 15 seconds, the subject leans over one hand and picks up the opposite hand reaches over to touch hands and then returns the hand to the starting position.

After the instructions and demonstration, every subject performed a familiarization task, performing few repetitions of hand touches. Verbal cues were given during familiarization when necessary.

Then, for data collections, each volunteer performed three trials of 15 seconds. The time counting started when the second examiner said “GO” and stopped when the same examiner said “STOP”. A time rest of 45 seconds was established between repetitions, because a work/rest ratio of 1:3 was suggested as appropriate for avoiding fatigue effects in the performance during a short duration and relative high intensity test, such as CKCUES Test [2]. If the subject was unable to perform properly the test, he/she was stopped and after 45 seconds of rest another repetition was tried. Acceptable male test repetition was defined as fully test complete with back flat, did not touch down the floor with knee(s), kept his weight-bearing upper extremity perpendicular to the floor and over his hands, and keep his/her feet as in the initial position. Females had an acceptable repetition when keeping back flat, weight-bearing upper extremity perpendicular to the floor and over his hands.

Rating Numeric Rating Scale (RNRS) [16] was applied to assess pain before and after experimental procedure of data collecting. Pain was analyzed to observe if test could cause pain, in which level and, also to report if a specific level of pain could prevent the test accomplishment. In the second session, experimental procedures were repeated to obtain data to estimate test–retest reliability, with exception of physical examination.

### Data analysis

The CKCUES test was proposed [8] to provide three scores: 1) *Number of touches score*: representing the number of touches that subjects can perform in 15 seconds. 2) *Normalized score*: obtained dividing the number of touches by subject height and; 3) *Power score*: obtained by multiplying the average number of touches by 68% of subject’s body weight in kilograms (percentage that corresponds to the weight of the arms, head and trunk) divided by 15 (elapsed test time in seconds).

Intrasession and intersession reliability of CKCUES test were calculate using values scores obtained from the last three of four repetitions in both sessions. The intersession reliability of CKCUES Test was determined for number of touches, normalized score and power score using mean values of the last three repetitions between test and retest session. The intrasession reliability of CKCUES Test was determined for number of touches score of test and retest. Number of touches, normalized score and power score mean values were compared among groups within the same gender in both sessions.

Standard Measurement Error (SEM) was calculated for comparisons of scores of CKCUES Test between groups of the same gender. This analysis was determined aiming to estimate how reliably the scores of CKCUES Test estimate an “true score” that could be obtained for a subject if the scores measures rightly, without error [13]. The Minimal Detectable Change (MDC) also was calculated for comparisons between groups of the same gender. MDC is the minimal amount of change required for the CKCUES Test score exceeds the measurement error; that is to determine the smallest amount of change that could be considered relevant to detect a difference between 2 measurements of same score.

Pain measurement was obtained by Visual Numeric Rating Scale (VNRS). Rating 0 represents no pain, rating 1–3 represents mild pain; rating 4–6 represents moderate pain, and rating 7–10 represents severe pain [15]. VNRS ratings of pain were compared pre and post test performing for each session. Pain scores will be showed in percentile, for each session and for each sample.

### Statistical analysis

Reliability was assessed by using intraclass correlation coefficient (ICC) with 95% confidence interval. The intersession reliability of number of touches, normalized score, and power score was assessed using ICC_2,3_ model (two-way random effect model analysis of variance). To determine the intrasession reliability of number of touches ICC_2,1_ model (two-way random effect model analysis of variance) was chosen for analysis [17]. ICC values of 0.75 and above represent good reliability, values between 0.40 and 0.74 represent moderate reliability and those below 0.40 indicate poor reliability 0.75 [18]. Statistical analyses were conducted using SPSS Version 16.0.

Standard Error Measurement (SEM) and Minimum Detectable Change at 95% of Confidence Interval (CI) were determined for each test score by the formulas:

$\mathrm{SE}M_{95}=\mathrm{SD}*\sqrt{\left(1-\mathrm{IC}C_{\mathrm{test}-\mathrm{retest}}\right)}$, where SD is the standard deviation of mean at baseline; and ICC _(2,3)_ value was derived from test-retest reliability [13]. The SEM was multiplied by the *z* value associated with the 95% CI (z = 1.96) to achieve 95% confidence level.

$\mathrm{MD}C_{95}=1.96*\mathrm{SE}M_{95}*\sqrt{2}$, where SEM is the standard error of measurement [19]. The 95% CI was calculated for the MDC, which is the statistically minimal amount of change required in CKCUES Test score to be 90% confident that true change has occurred.

A one-factor Analysis of Variance was chosen to compare scores of CKCUES Test between SIS, sedentary and recreationally athletes for upper extremity sport specific groups within same gender. The Shapiro-Wilk test was used for post hoc analysis when differences were found. The level of significance was set at 5%.

## Results

Descriptive data of number of touches and normalized score showed that values for healthy groups were considered inside the CKCUES Test reference scores (Table 2). Scores of SIS samples were lower than reference scores values (Table 2). All scores obtained of healthy female and healthy male upper extremity sport-specific athletes were greater than its correspondent gender of SIS group. With exception of power score compared among females groups of sedentary and healthy upper extremity sport specific athletes, all scores were greater where compared between active and healthy groups. When sedentary males and active males were compared, there were not found differences between scores (Table 3).

**Table 2:** Test and retest mean (and standard deviation) of CKCUES Test scores and reference values

**CKCUES test scores**						
	**Number of touches**		**Power**		**Normalized score**	
**Groups**	**Test**	**Retest**	**Test**	**Retest**	**Test**	**Retest**
**Sedentary male (n=20)**	22.67 (3.75)	25.30 (3.68)	83.43 (14.66)	93.37 (16.10)	0.33 (0.06)	0.37 (0.06)
**Sedentary female (n=20)**	24.58 (4.48)	28.47 (4.96)	62.93 (14.60)	72.87 (17.05)	0.39 (0.07)	0.45 (0.08)
**Active**^**¥ **^**Male (n=20)**	24.78 (3.19)	27.13 (3.15)	84.47 (11.59)	92.78 (13.92)	0.40 (0.05)	0.39 (0.05)
**Active**^**¥ **^**Female (n=20)**	27.97 (3.84)	31.97 (4.47)	72.55 (12.90)	83.06 (16.23)	0.44 (0.06)	0.51 (0.08)
**Male SIS**^**£ **^**(n=13)**	10.10 (3.31)	11.82 (2.68)	38.59 (14.46)	45.02 (12.22)	0.15 (0.05)	0.18 (0.04)
**Female SIS**^**£**^**(n=15)**	12.20 (3.64)	13.73 (3.41)	37.63 (12.54)	42.33 (12.49)	0.20 (0.06)	0.23 (0.06)
**Male reference values**	**18.5**		**150**		**0.26**	
**Female reference values**	**20.5**		**135**		**0.31**	

^¥^Active = young healthy male or female recreationally active for upper extremity sport-specific.

^£^SIS = subacromial impingement syndrome.

**Table 3:** CKCUES Test scores [mean (standard deviation)] for comparisons within gender groups

	**CKCUES test**					
	**Number of touches**		**Power**		**Normalized score**	
**Groups**	**Test**	**Retest**	**Test**	**Retest**	**Test**	**Retest**
**Sedentary male (n=20)**	22.67 (3.75)*	25.30 (3.68)*	83.43 (14.66)*	93.37 (16.10)*	0.33 (0.06)*	0.37 (0.06)*
**Active**^**¥ **^**Male (n=20)**	24.58 (4.48) ^○^	28.47 (4.96) ^○^	62.93 (14.60) ^○^	72.87 (17.05) ^○^	0.39 (0.07) ^○^	0.45 (0.08) ^○^
**Active**^**¥ **^**Female (n=20)**	27.97 (3.84) ^§■^	31.97 (4.47) ^§■^	72.55 (12.90) ^■^	83.06 (16.23) ^■^	0.44 (0.06) ^§■^	0.51 (0.08) ^§■^
**Sedentary female (n=20)**	24.78 (3.19) ^§^&	27.13 (3.15) ^§^&	84.47 (11.59) &	92.78 (13.92) &	0.40 (0.05) ^§^&	0.39 (0.05) ^§^&
**Male SIS**^**£ **^**(n=13)**	10.10 (3.31)* ^○^	11.82 (2.68)* ^○^	38.59 (14.46)* ^○^	45.02 (12.22)* ^○^	0.15 (0.05)* ^○^	0.18 (0.04)* ^○^
**Female SIS**^**£ **^**(n=15)**	12.20 (3.64) &^■^	13.73 (3.41) &^■^	37.63 (12.54) &^■^	42.33 (12.49) &^■^	0.20 (0.06) &^■^	0.23 (0.06) &^■^

^¥^Active = young healthy male or female recreationally active for upper extremity sport-specific ^■^.

^£^SIS = subacromial impingement syndrome.

^§^significant difference between sedentary and young recreationally healthy female.

& significant difference between sedentary and SIS female.

*significant difference between sedentary and SIS male.

^■^significant difference between SIS female and young recreationally healthy female.

^○^significant difference between SIS male and young recreationally healthy male.

The intrassesion reliability of CKCUES Test for all samples showed excellent intraclass correlation coefficient (ICC > 0.75) for number of touches, power score and normalized score. Intersession reliability of CKCUES test for all scores also showed excellent values of ICC (ICC >0.75) for all samples (Table 4).

**Table 4:** Intraclass correlation coefficients (confidence intervals of 95%) for CKCUES Test score intrasession and intersession reliabilities

	**Intersession reliability**			**Intrasession reliability**	
**Groups**	**Number of touches**	**Power**	**Normalized score**	**Number of touches**	**Number of touches**
	**Test-retest**	**Test-retest**	**Test-retest**	**Test**	**Re-test**
	**ICC**_**2,3 **_**(IC95%)**	**ICC**_**2,3 **_**(IC95%)**	**ICC**_**2,3 **_**(IC95%)**	**ICC**_**2,1 **_**(IC95%)**	**ICC**_**2,1**_**(IC95%)**
**Sedentary male (n=20)**	0.96	0.96	0.96	0.96	0.96
	(0.89;0.98)	(0.90;0.99)	(0.89;0.98)	(0.92;0.98)	(0.92;0.98)
**Sedentary female (n=20)**	0.92	0.96	0.92	0.96	0.97
	(0.80;0.97)	(0.89;0.98)	(0.81;0.97)	(0.92;0.98)	(0.93;0.99)
**Active**^**¥ **^**Male (n=20)**	0.89	0.84	0.90	0.93	0.95
	(0.71;0.96)	(0.58;0.94)	(0.75;0.96)	(0.95;0.99)	(0.89;0.98)
**Active**^**¥ **^**Female (n=20)**	0.85	0.82	0.87	0.90	0.95
	(0.62;0.94)	(0.55;0.93)	(0.67;0.95)	(0.90;0.99)	(0.90;0.98)
**Male SIS**^**£ **^**(n=13)**	0.91	0.93	0.92	0.96	0.97
	(0.70;0.97)	(0.75;0.98)	(0.72;0.97)	(0.88;0.99)	(0.92;0.99)
**Female SIS**^**£**^**(n=15)**	0.93	0.94	0.94	0.86	0.92
	(0.78;0.98)	(0.81;0.98)	(0.81;0.98)	(0.70;0.98)	(0.82;0.97)

^¥^Active = young healthy male or female recreationally active for upper extremity sport-specific.

^£^SIS = subacromial impingement syndrome.

Number of touches SEM for groups ranged from 1.45 to 2.76 touches, and MDC95% ranged from 2.05 to 3.91 touches. For power scores, SEM ranged from 6.02 to 20.03, and MDC from 8.52 to 28.32. Normalized score SEM ranged from 0.02 to 0.04, and MDC from 0.03 to 0.06 (Table 5).

**Table 5:** Standard error of measurement and minimal detectable change of each CKCUES Test score

**Groups**	**Number of touches**		**Power**		**Normalized score**	
	**SEM**_**95**_	**MDC**_**95**_	**SEM**_**95**_	**MDC**_**95**_	**SEM**_**95**_	**MDC**_**95**_
**Sedentary male (n=20)**	1.45	2.05	12.58	17.79	0.02	0.03
**Sedentary female (n=20)**	2.43	3.43	12.94	18.30	0.04	0.05
**Active**^**¥ **^**Male (n=20)**	2.0	2.82	20.03	28.32	0.03	0.04
**Active**^**¥ **^**Female (n=20)**	2.76	3.91	12.94	18.30	0.04	0.06
**Male SIS**^**£ **^**(n=13)**	1.95	2.76	7.50	10.61	0.03	0.04
**Female SIS**^**£**^**(n=15)**	1.89	2.67	6.02	8.52	0.03	0.04

^¥^Active = young healthy male or female recreationally active for upper extremity sport-specific.

^£^SIS = subacromial impingement syndrome.

SEM_95_ = standard error of measurement.

MDC_95_ = minimal detectable change.

Healthy subjects reported no shoulder pain before performing the test in both sessions. After test, mild pain was reported by males sport specific recreational athletes (25% of subjects in test and 15% in retest), by females sport specific recreational athletes (35% of subjects in test and 25% in retest), and by sedentary females (10% in test and 20% in retest). Sedentary males reported mild (20% in test and 25% in retest) and moderate pain (10% in test and 5% in retest) after test.

For samples with SIS, 96.4% of subjects reported severe and moderate pain before test in both sessions. After test, 86.7% of females in the test session and 80% in the retest session remained with pain in moderate and severe levels and 16.7% reported pain relief after test session (13.3% in test and 20% in retest), changing their pain level to a lower category.

## Discussion

The purpose of this study was to evaluate the test-retest reliability of Closed Kinetic Chain Upper Extremity Stability Test- CKCUES test scores. Results showed excellent values of ICC for intersession reliability of number of touches score. Intrasession reliability of test also showed excellent (ICC ≥ 0.75) values scores for all samples. The results support the reliability of CKCUES Test as a complementary outcome measure for evaluating shoulder functional condition in healthy sedentary, healthy active and SIS subjects.

Results of intrasession reliability of this research are in accordance to Goldbeck & Davies, the only study found in the literature about CKCUES Test reliability. However, Goldbeck & Davies assessed just reliability of number of touches score in a sample of male recreational athletes. Moreover, there were not found studies about CKCUES Test reliability in females, sedentary subjects and in a sample with shoulder injury. Thus, some results of this study were not possible to be compared with other researches.

There was found in the literature other research that has determined reliability of other closed kinetic chain performance tests for upper extremity, with excellent values of test-retest reliability [1]. However, that research also has not evaluated subjects with shoulder dysfunctions or injuries. Moreover, that test includes an equipment to sample quantitative data. Thus, we believe our results could be important for sportive and clinical assessment for two reasons. First because CKCUES Test is a low cost test with no need of an equipment to measure the scores, and second because the reliability of the test was determined for samples with different levels of physical conditioning and also in sample with SIS. Thus, we believe clinicians and athletic trainers can choice this test to first evaluations and to follow-ups of upper extremity performance.

Number of touches and normalized score values obtained in this research were greater than the reference values for CKCUES Test [8]. However, it is important to consider that from the original reference there is not a range of values, but only a unique value for each score that could be considered as reference. Thus if a person has his/her score lower than reference values, scores of test can be improved. Otherwise, if scores are greater than references values, a comparison of those score could be done before and after a specific training as parameter of evaluation. Anthropometric characteristics of samples could have influenced these results, mainly because height and weight are used in the formulas for calculating normalized score and power score, respectively.

A possible justification regarding wider confidence interval of intersession reliability values of ICC of recreational athletic samples and SIS samples for all scores could be the variability of weight and height among subjects at the same group. Despite the excellent reliability for number of touches, it is important to consider that power and normalized score are dependent of the subject’s anthropometric variable. As adults composed the samples those variations is more likely to be from their body weight changes.

Moreover, considering the difference in the subject’s wingspan, i.e. the distance between fingertip to fingertip of middle fingers with the arms spread, the fact of this test consider a unique value of 36-inches (91,44 cm) distance between hands, independent of subject’s height [8], could limit the comparisons of performance among subjects. For example, a taller person could perform the test faster than one smaller due the larger wingspan, which could result in a greater score of number of touches, and consequently, in a greater value of normalized score and power score. Thus, when normalized score and power score are analyzed, anthropometric variables are considered in the score estimates.

On the other hand, there is another possibility to diminish the influence from the anthropometric characteristics of the subject’s height on the number of touches score by setting the distance between hands as a percentage of the total size of the subject’s wingspan or as a distance between scapular acromions. However, further studies are necessary to analyze if those changes in the CKC test are feasible, create reliable scores and safety biomechanics for patients with shoulder dysfunctions.

To date, no studies analyzing the SEM and MDC of scores of CKCUES Test were found. However, it is important to have knowledge about what is the minimal difference in the scores of an evaluation tool between revaluation sessions that could be considered as a real improvement with no error [13,14]. Thus, our results could guide clinicians and athletic trainers with these values for subjects with and without SIS and with different levels of physical activity lifestyle.

Considering values of SEM and MDC for females groups, changes between sessions could be considered as a true change when number of touches score exceeds 4 touches for a sedentary, 4 touches for a active and 3 for a SIS person. In groups of males, changes between sessions could be considered as a true change when number of touches score exceeds 2 touches for a sedentary, 3 for active and 3 for a SIS person. With those number of touches changes, normalized score and power score also will be changed. Thus, those number of touches values could be considered as the minimal change between CKCUES Test evaluations that could be considered as a real change of improvement.

Some healthy subjects reported shoulder pain after test, even with no pain reporting before the test. A possible justification could be the fact that CKCUES Test is a high level performance test, which can cause a high demand over shoulders. Lastly, based on reliability results from samples with SIS, shoulder pain was not an impediment to those subjects performs the test, independently of rating level. In samples with SIS, a greater level of pain post test compared to pre test was expected before by the same justification. Thus, clinicians should have care when the test is considered in the initial clinical evaluation of a subject with shoulder injury. Care also should be taken when the test is being performed. If a subject shows an incorrect body positioning or some compensatory movements, or if the subject report pain during the test, an interruption might be necessary, since the axial load applied to the arm 90 degrees elevate in anterior flexion is close to body weight when the subject is touching hands in the end of the swing phase.

Results showed that CKCUES Test is a reliable tool to evaluate upper extremity function in sedentary and young male or female recreationally active subjects and also in subjects with SIS. However, this study has some limitations, such as subjects without shoulder injury are from a young population, elite athletes were not included, and only with SIS participants represented shoulder dysfunction in our sample. This way, the results of this study should be carefully analyzed when extended to other populations.

## Conclusion

Results showed that CKCUES Test is a reliable tool for evaluating upper extremity functional activity in sedentary and upper extremity sport specific males and females and also in subjects with subacromial impingement syndrome. Results also showed that a change of at least 3 touches for sedentary active and subacromial impingement syndrome males, and at least 3 touches for subacromial impingement syndrome females and 4 touches for active and sedentary female is necessary to be considered as an improvement in the CKCUES Test scores.

### Implications for physiotherapy practice

Different from lower extremity, upper extremity performance test are not profuse. Any additional tool to asses upper extremity performance is important for practitioners if it has clinical or sportive meaningful information, if it could be simply fitted to clinical and sportive settings with low cost and, if it has clinimetric proprieties tested, as Reliability, Standard Error Measurement and Minimum Detectable Change. In this way, CKCUES Test showed to be a valuable performance test for both healthy and subacromial impingement syndrome subjects with different levels of physical activity lifestyle. Thus, this study had contributed to practice presenting how much reliable is CKCUES Test and how to interpret the change in the Number of Touches Scores when applying it as a performance follow-up test.

## Competing interests

The authors declare that they have no competing interests.

## Authors’ contributions

HTT: participated in the design of the study, data collecting, data analysis and draft the manuscript. JM: participated in the design of the study, data collecting, performed the statistical analysis, and draft the manuscript. GCS: participated the design of the study and recruitment of the subjects. ASO: participated in the design of the study and draft the manuscript. All authors read and approved the final manuscript.

## Pre-publication history

The pre-publication history for this paper can be accessed here:

http://www.biomedcentral.com/1471-2474/15/1/prepub
